# 16S rRNA Gene Amplicon Sequence Data from Chicken Cecal Feces from Vietnam and Thailand

**DOI:** 10.1128/MRA.00781-19

**Published:** 2019-08-08

**Authors:** Nachiko Takeshita, Hyunjung Kim, Kringkrai Witoonsatian, Mari Tohya, Tấn Hùng Võ, Nuchjaree Boonyong, Thị Phương Bình Nguyễn, Ichiro Nakagawa, Nattakan Meekhanon, Ngọc Hải Nguyễn, Tsutomu Sekizaki

**Affiliations:** a Research Center for Food Safety, Graduate School of Agricultural and Life Sciences, The University of Tokyo, Tokyo, Japan; b Department of Farm Resources and Production Medicine, Faculty of Veterinary Medicine, Kasetsart University, Nakhon Pathom, Thailand; c Pathogenic Microbe Laboratory, Research Institute, National Center for Global Health and Medicine, Tokyo, Japan; d Veterinary Diagnosis Laboratory, Faculty of Animal Science and Veterinary Medicine, Nong Lam University, Ho Chi Minh City, Vietnam; e Department of Veterinary Technology, Faculty of Veterinary Technology, Kasetsart University, Bangkok, Thailand; f Department of Microbiology, Graduate School of Medicine, Kyoto University, Kyoto, Japan; g Department of Contagious Diseases and Public Health, Faculty of Animal Science and Veterinary Medicine, Nong Lam University, Ho Chi Minh City, Vietnam; Georgia Institute of Technology

## Abstract

Here, we report 16S rRNA amplicon sequence data from chicken cecal feces from Vietnam and Thailand. Lachnospiraceae, Ruminococcaceae, and Bacteroidaceae were dominant in cecal feces microbiota.

## ANNOUNCEMENT

Chicken is a common domesticated animal that is important for food production worldwide. Production of healthy livestock helps ensure a safe food supply, and a healthy microbial community in the gastrointestinal tract underpins the links between diet and health in chickens (1). In the gastrointestinal tract, the cecum is the most densely populated and diverse bacterial habitat, and various roles have been suggested for the cecal microbiota (2, 3). Breeding systems and species of chickens differ among countries, and the gut microbiota may reflect these differences. In this study, we analyzed the microbiota of dropped cecal feces of chickens in Vietnam and Thailand because the microbiota of dropped cecal feces is very similar to that of cecal contents (3).

In August 2017, cecal feces samples from 7 clinically healthy chickens (Luong Phuong breed) were collected from two broiler houses in Củ Chi district, Vietnam. The houses were windowless, and each accommodated 70,000 to 80,000 birds. In November 2017, cecal feces samples from 7 clinically healthy chickens (Arbor Acres breed) were collected from two broiler houses in Nakhon Pathom province, Thailand. An evaporative cooling system was used, and 10,000 birds were accommodated in each house.

Cecal feces dropped on the floor were immediately collected using a 150-mm polypropylene spatula (As One, Osaka, Japan). Each sample was immersed independently in 500 μl of RNA*later* stabilizing solution (Thermo Fisher Scientific, Waltham, MA, USA) in a conical tube. The tubes were stored at –20°C until use. Frozen samples were thawed and washed as previously described (4), and then the DNA in the samples was extracted using a PowerFecal DNA isolation kit (Qiagen, Hilden, Germany). To increase the extraction efficiency, zirconia beads (Toray, Tokyo, Japan) were used instead of the beads in the kit (4).

The V3 and V4 regions of the 16S rRNA genes in the extracted DNA were amplified with S-D-Bact-0341-b-S-17 (5´-TCGTCGGCAGCGTCAGATGTGTATAAGAGACAGCCTACGGGNGGCWGCAG-3´) and S-D-Bact-0785-a-A-21 (5´-GTCTCGTGGGCTCGGAGATGTGTATAAGAGACAGGACTACHVGGGTATCTAATCC-3´) primers (5) including an Illumina overhang adapter sequence (Illumina, San Diego, CA, USA). Sequencing was performed using the 2 × 300-bp paired-end method on the MiSeq platform with a MiSeq v3 reagent kit (Illumina), and a total of 2,436,687 raw reads were generated from 28 samples. FASTQ reads were processed using the IM-TORNADO pipeline (v2.0.3.2) (6), with default parameters, except that the following parameters were used in Trimmomatic: LEADING:20, TRAILING:20, and MINLEN:180. The reads were filtered for quality using Trimmomatic and merged using scripts in the pipeline. The pipeline used mothur (7) for operational taxonomic unit (OTU) clustering at 100% sequence identity and employed a k-mer-based approach for taxonomy assignment using the Ribosomal Database Project (RDP) naive Bayesian classifier (8) with a threshold of 80% bootstrap confidence. Each OTU was assigned at the family level against the RDP database (9) at 97% sequence identity. Ethics approval for the study was granted by the Animal Research Committee of The University of Tokyo.

Taxonomic classifications at the family level showed that *Lachnospiraceae* (17.2 to 42.6%), *Ruminococcaceae* (15.8 to 44.2%), and *Bacteroidaceae* (3.4 to 15.7%) were dominant, and these three bacterial taxa accounted for almost 50% of the microbiota, in agreement with a previous report (10).

### Data availability.

Data sets generated by 16S rRNA gene amplicon sequencing in this study have been deposited in the DNA Data Bank of Japan (DDBJ)/SRA under accession number DRA008534.
